# Anti-IH in myelodysplastic syndrome

**DOI:** 10.1016/j.plabm.2025.e00468

**Published:** 2025-03-22

**Authors:** Ketsaraporn Wongba, Pornlada Nuchnoi, Chotiros Plabplueng, Charuporn Promwong

**Affiliations:** a Department of Blood Bank and Transfusion Medicine, Sunpasitthiprasong Hospital, Ubon Ratchathani, Thailand; b Department of Clinical Microscopy, Faculty of Medical Technology, Mahidol University, Bangkok, Thailand; c Center for Research Innovation and Biomedical Informatics, Faculty of Medical Technology, Mahidol University, Bangkok, Thailand

**Keywords:** Anti-IH, Cold autoantibody, Cold agglutinin, Hemolytic transfusion reaction, Myelodysplastic syndrome, ABH antigen

## Abstract

**Background:**

Anti-IH exhibits complex specificity, strongly reacting with cells expressing both H and I antigens at cold temperatures. Its clinical significance has been increasingly recognized, particularly in patients with hematologic conditions such as myelodysplastic syndrome (MDS) and chronic myelomonocytic leukemia (CMML).

**Case report:**

We present an 83-year-old Thai female with MDS who was transfusion-dependent. She presented with severe anemia requiring an urgent transfusion. The patient was group A RhD-positive. Antibody screening and identification using column agglutination technology (CAT) showed weak polyagglutination. Auto-control and direct antiglobulin tests (DAT) were negative. Red cell typing showed absence of H antigen and presence of A1 antigen. Further testing with cord O cells revealed no agglutination, confirming the A1 blood group with anti-IH. Antibody screening and identification studies showed cold-reactivity, with weak reactivity at 37 °C and in the AHG phase. Crossmatching with two group A leukocyte-poor red cells was compatible, and transfusion was uneventful.

**Conclusion:**

This is the first reported case of anti-IH in a Thai patient. Anti-IH may complicate pre-transfusion testing and mask alloantibodies, necessitating careful interpretation and confirmatory testing to prevent transfusion-related complications.

## 1 Introduction

The cold alloantibodies and autoantibodies frequently identified in clinical practice are commonly directed against the Ii blood group collection. The ABH and Ii antigens are structurally related and co-expressed on red blood cells. The cold allo/autoantibody that targets both H and I antigens simultaneously expressed on the same cell is known as anti-IH. Anti-IH can complicate serologic testing, particularly antibody screening and identification, at room temperature. This antibody is most commonly found in individuals with blood groups A1, B, or A1B [1]. While anti-IH typically demonstrates a preference for cold temperatures and rarely causes clinically significant hemolysis, it has been implicated in hemolytic transfusion reactions (HTRs) in patients with sickle cell disease [1] and hematologic malignancies [2]. Moreover, cases of anti-IH-mediated HTRs have also been reported in individuals who received A2 red cells, even in the absence of an underlying hematologic condition [3]. In this report, we describe the identification of anti-IH in a patient with myelodysplastic syndrome (MDS) and A RhD-positive blood group during pre-transfusion testing. Prompt recognition of the serologic characteristics of anti-IH and access to additional confirmatory testing are essential for accurate diagnosis and optimal transfusion management.

## 2 Case presentation

### 2.1 Clinical history

An 83-year-old Thai female patient with a blood group of A RhD-positive had been diagnosed with myelodysplastic syndrome (MDS) in 2017. The patient had a history of multiple blood transfusions, as summarized in Table 1. Her ongoing medications included 0.5 tablets of prednisolone (5 mg) and a daily dose of vitamin B/folic acid. Additionally, she was prescribed calcium carbonate (CaCO_3_) and vitamin D2 for the management of osteoporosis. On September 19, 2024, the patient attended the hospital for an MDS follow-up. Laboratory results revealed a hemoglobin level of 6.8 g/dL and hematocrit level of 23.6 %, indicative of severe anemia. Consequently, two units of leukocyte-poor packed red blood cells (LPRC) were requested.

**Table 1:** Summary of the patient's transfusion history, antibody screening results, and hemoglobin and hematocrit levels before and after transfusion.

Date	Antibody screening (CAT)	Type and unit of blood components	Hemoglobin (g/dL)		Hematocrit (%)	
			before transfusion	after transfusion	before transfusion	after transfusion
2018-05-03	Negative	1 LPRC	7.6	8.8	23.8	29.6
2018-06-20	Negative	2 LPRC	6.9	9.3	21.9	29.4
2019-03-07	Negative	1 LPRC	7.7	9.6	25.1	ND
2023-10-19	Negative	2 LPRC and 2 pooled LDPC	7.8	8.4	25.8	ND
2024-09-19	Positive	2 LPRC	7.5	9.4	23.6	30.8

CAT: column agglutination test, LPRC: leukocyte-poor packed red blood cells, LDPC: leukocyte depleted platelet concentrate, ND: not determined.

### 2.2 Laboratory examination

Forward and reverse typing for ABO and RhD testing confirmed the patient's blood group as A and RhD-positive, respectively. During pre-transfusion testing, antibody screening revealed weak reactivity with O_3_ screening cells and negative for O_1_ and O_2_ cell (O_1_, O_2_: Grifols, O_3_: NBC, Thai Red Cross) using the column agglutination test (CAT) (Ortho Clinical Diagnostics, USA). Antibody screening using the conventional tube test (CTT) showed strong positivity at 4 °C and room temperature (RT), while results were nonreactive at 37 °C and in the anti-human globulin (AHG) phase (Table 2). Antibody identification demonstrated strong pan-reactivity at the immediate spin (IS) phase and at room temperature, with progressively weaker reactions at 37 °C and AHG) phase, indicating a cold-reactive autoantibody. Both the auto-control and direct antiglobulin test (DAT) were negative. Further testing revealed that the patient's red blood cells were nonreactive with a monoclonal anti-H (Anti-H: murine moAb: Thai Red Cross; Thailand) but positive for A_1_ antigen (Anti-A1: murine moAb: Thai Red Cross; Thailand). The patient's plasma showed no agglutination with five O red cells from cord and adult A1 cells but exhibited a 2+ reaction with adult A2 cells, as summarized in Table 3. Crossmatching of group A donor red cells with the patient's plasma was serologically compatible in the AHG phase using CAT. Consequently, two units of group A leukocyte-poor red cells (LPRC) were transfused without any adverse transfusion reactions.

**Table 2:** Antibody screening test.

Antibody Screening	CTT				
	4 °C	RT	37 °C	AHG	CCC
O1	4+	3+	0	0	2+
O2	4+	3+	0	0	2+
O3	4+	3+	0	0	2+

RT: room temperature, AHG: Anti-human globulin, CTT: conventional tube test, CCC: Coombs control cell.

**Table 3:** Antibody identification results using conventional tube test (CTT) and column agglutination technology (CAT).

Antibody identification	CTT				CAT
	RT	37C	AHG	CCC	AHG
O1	4+	2+	0	2+	0.5+
O2	4+	1+	0	2+	0.5+
O3	4+	2+	0	2+	0.5+
O4	4+	1+	0	2+	0.5+
O5	4+	2+	0	2+	0.5+
O6	4+	2+	0	2+	0.5+
O7	4+	2+	0	2+	0.5+
O8	4+	2+	0	2+	0.5+
O9	4+	2+	0	2+	0.5+
O10	4+	1+	0	2+	0.5+
O11	4+	1+	0	2+	0.5+
Auto control	0	0	0	2+	0
Oi_1_ - Oi_5_ (cord RBC)	0				
A1	0				
A2	2+				

AHG: Anti-human globulin, CAT: column agglutination technology, CCC: Coombs control cell, CTT: conventional tube test, RT: room temperature.

## 3 Discussion

During antibody screening using the CAT, weak agglutination was observed in the AHG phase. Antibody identification demonstrated pan-reactivity at RT and 37 °C, with a negative auto-control, suggesting the presence of a cold-reactive antibody. Anti-IH was suspected and subsequently confirmed through further testing, which revealed no reactivity with A1 cells and cord O cells (I-) but strong reactivity with adult O cells (I+) at room temperature. The absence of the I antigen in cord O cells, contrasted with its abundance in adult O cells, was instrumental in identifying anti-IH. This additional serologic investigation highlighted the presence of anti-IH in the patient’s plasma, confirming it as the most probable antibody in this case.

The H antigen serves as the precursor for the synthesis of A and B antigens, with expression levels decreasing in the order: O > A2 > B > A2B > A1 > A1B [4]. The patient was identified as having the A1 blood group. The low reactivity observed with the patient’s own red blood cells (negative auto-control) aligns with the low H antigen expression characteristic of individuals with the A1 blood group. Despite a history of multiple transfusions with group A leukocyte-poor red cells (LPRC) or leukocyte-depleted red cells (LDRC), the patient did not develop alloantibody.

The antibody screening results in Table 2 demonstrate that the antibody exhibited strong reactivity at lower temperatures, with a 3+ reaction at room temperature (RT). Similarly, the antibody identification results in Table 3 reveal a comparable pattern, albeit with an even stronger 4+ reaction at RT. This pattern suggests the presence of cold-reactive autoantibodies, which are known to interfere with routine blood bank testing. The inconsistent results between the 37 °C antibody screening and antibody investigation can occur due to the nature of testing. Furthermore, the anti-human globulin (AHG) phase, which is specific for IgG detection, yielded negative results, reinforcing the hypothesis that the detected antibody is of the cold type rather than a warm IgG antibody.

The observed discrepancy between the CTT and CAT methods, particularly the negative anti-human globulin (AHG) phase in CTT compared to the positive CAT results, can be attributed to the higher sensitivity of the CAT. As reported in the study [5], CAT consistently yielded titers approximately 2.5 times higher than CTT (mean titer 96.4 ± 225 vs. 38.5 ± 96.6), reflecting its ability to detect weaker agglutination reactions that CTT may miss. This heightened sensitivity of CAT arises from its gel-based matrix, which stabilizes antibody-antigen complexes more effectively than the liquid-phase methodology of CTT. The negative AHG phase in CTT suggests that the anti-IH detected by CAT is unlikely to be of the IgG class, as IgG antibodies typically require the AHG phase for detection. Instead, the results from CAT strongly imply that the detected anti-IH is of the IgM class, which does not require the AHG phase for agglutination and can be more easily captured by CAT due to the higher sensitivity of the gel matrix. This suggests that CAT has a unique advantage in identifying cold-reactive antibodies like anti-IH, which may be missed by less sensitive methods like CTT. The increased sensitivity of CAT also underscores its clinical utility in the context of ABO-incompatible kidney transplants. By detecting antibodies at lower concentrations or with weaker avidity, CAT ensures that clinically significant antibodies are not overlooked, enabling more accurate assessment and management of antibody-mediated risks. These findings highlight the need for method-specific thresholds and further standardization to incorporate the sensitivity differences into clinical practice effectively.

A prewarming technique was not utilized in this study While this may have limited the study's ability to detect potential clinically significance antibody that can potentially react with red blood cell at body temperature. Our findings are based on standard serologic techniques, including CAT and CTT using room temperature saline. We recognize that the application of prewarming technique would have provided more precise information regarding the antibody’s reactivity at 37 °C. Prewarming both the patient plasma and red cells to 37 °C before testing, as described previously [6], minimizes interference from cold-reactive antibodies and ensures adherence to strict temperature control during testing. The absence of prewarming represents a limitation of this investigation. Future studies should incorporate these measures to facilitate a more comprehensive and robust evaluation of relevant antibodies, including anti-IH. Despite this limitation, the lack of reactivity in the antiglobulin phase indicates that the anti-IH identified in this study is unlikely to exhibit clinically significant activity at body temperature. Future studies will seek to address this limitation by incorporating prewarming technique and conducting thermal amplitude studies to provide a more accurate interpretation of the results.

The anti-IH identified in this study exhibited weak reactivity at 37 °C, indicating potential clinical hemolysis. However titration studies are essential to better understand the clinical significance of anti-IH [7], and future study should incorporate the analyses. Although anti-IH was traditionally considered clinically insignificant due to its preference for cold temperatures and low titers [3], recent reports have implicated it in acute HTR in patients with sickle cell disease (SCD) [1] and MDS [2,8]. With an increasing number of reported cases, anti-IH should now be regarded as a clinically significant antibody until clinical significance can be established. When possible, crossmatch-compatible blood should be transfused. Its detection during pre-transfusion testing, as demonstrated in this case, is critical. Anti-IH is most commonly found in blood groups A_1_, B, and A_1_B. Due to the low H antigen expression in these groups, transfusion with A_2_ or O blood group red cells in patients with anti-IH may result in severe adverse events, including acute hemolysis, as observed in A1 patients transfused with A_2_ red blood cells [3]. Therefore, ABO group-specific or subgroup-compatible red blood cells are recommended to ensure compatibility in transfusion management for patients with anti-IH, as these options minimize the risk of hemolytic reactions caused by incompatible H antigen exposure.

In this study, anti-IH was detected in a patient with MDS and a history of multiple transfusions for the treatment of severe anemia. The patient had previously received four transfusions of group A LPRC/LDRC over several years without the production of alloantibodies, as antibody screening tests were negative. However, during pre-transfusion testing for the fifth transfusion, eight years after the MDS diagnosis, anti-IH was identified. MDS, a group of myeloid neoplasms characterized by cytopenia, may influence ABH and I antigen expression [9]. Alterations in these antigens, particularly reductions in ABH or I antigen levels, are frequently observed in hematologic malignancies [10], including decreased A antigen expression in patients with acute myeloid leukemia (AML) [[10], [11], [12]]. The precise immune cell subset responsible for breaking tolerance remains unclear in this context. While altered antigen expression and immune dysregulation associated with malignancies have been documented in some cases, a definitive immunological mechanism for anti-IH development in neoplastic conditions has yet to be established. Therefore, the clinically insignificant anti-IH are often naturally occurring [6].

While anti-IH is commonly a naturally occurring antibody with minimal clinical significance, its presence in this patient warrants further consideration due to the potential interplay between altered antigen expression and immune response, which may have implications for transfusion management. To our knowledge, this is the first reported case of anti-IH in a Thai patient with this specific combination of myeloid neoplasm. This combination makes the case distinct and contributes to the broader understanding of antibody development in such settings. We acknowledge the value of thermal amplitude and titration studies in characterizing the clinical significance of anti-IH. Unfortunately, the titration study was not performed in this case. However, given the absence of hemolysis or red cell destruction and the negative DAT, it is likely that the anti-IH in this patient is of limited clinical significance. Future studies in similar cases could benefit from incorporating thermal amplitude and titer analyses to provide a more comprehensive evaluation of antibody behavior and its potential clinical impact. Since this anti-IH is clinically insignificant and reactivity at 37 °C and AHG with A2 vs. A1 RBCs was not assessed, it is advisable to ensure the availability of A1+ RBCs for crossmatching to avoid delays in providing compatible blood if transfusion is required.

Anti-IH should be recognized as a clinically significant antibody until clinical significance can be established. Its presence in MDS patients underscores the need for further clarification on the relationship between changes in antigen expression and antibody detection. Additionally, more clinical case reports in neoplastic conditions are required to establish its relevance in laboratory practice.

## 4 Conclusion

Anti-IH is an autoantibody that exhibits strong reactivity at cold temperatures (4 °C) and weaker reactivity at 37 °C. It is increasingly identified in patients with hematologic diseases and has been implicated in hemolytic transfusion reactions. The presence of anti-IH can complicate the interpretation of pre-transfusion testing. To confirm its presence, specific panel cells, such as cord O cells (I-) should be tested with the patient’s plasma. For safe transfusion management, ABO group-specific or subgroup-compatible red blood cells remain the preferred recommendation for managing transfusions in patients with anti-IH.

## CRediT authorship contribution statement

**Ketsaraporn Wongba:** Formal analysis. **Pornlada Nuchnoi:** Conceptualization, Investigation, Writing – original draft, Writing – review & editing. **Chotiros Plabplueng:** Investigation, Writing – review & editing. **Charuporn Promwong:** Conceptualization, Formal analysis, Investigation, Writing – review & editing.

## Ethical issues

The work described in this report was conducted in compliance with the principles outlined in the Declaration of Helsinki. We follow the regulation of the ethic committee of Sunpasitthiprasong Hospital. All personal data were anonymized to ensure patient confidentiality. Clinical and laboratory data were utilized solely for the purposes of analysis and conceptualization, with no identifiable information included.

## Funding

No funding for this study.

## Declaration of competing interest

The authors declare the following financial interests/personal relationships which may be considered as potential competing interests: Dr.Pornlada Nuchnoi reports was provided by Mahidol University Faculty of Medical Technology Bangkok Noi Campus. If there are other authors, they declare that they have no known competing financial interests or personal relationships that could have appeared to influence the work reported in this paper.

## Data Availability

Data will be made available on request.
